# Contralateral recurrent laryngeal nerve palsy in revision anterior cervical discectomy and fusion (ACDF): A cautionary tale

**DOI:** 10.37796/2211-8039.1114

**Published:** 2021-03-01

**Authors:** Cheng Han Wu, Eugene Wei Ren Yang, Kelvin Kah Ho Lor

**Affiliations:** a Department of Orthopaedic Surgery, Khoo Teck Puat Hospital, Singapore; b Division of Neurosurgery, Department of Surgery, Khoo Teck Puat Hospital, Singapore

**Keywords:** anterior cervical spine surgery, recurrent laryngeal nerve palsy, revision surgery

## Abstract

Revision anterior cervical spine surgery has a higher risk of recurrent laryngeal nerve palsy (RLNP). We describe a unique case of an isolated RLNP contralateral to the side of the surgical approach in a patient who underwent revision anterior cervical discectomy and fusion (ACDF) for cervical myelopathy, and in whom pre-operative laryngoscopic evaluation had excluded a pre-existing occult RLNP. Scarring around the recurrent laryngeal nerve at the previous surgical site may have rendered it less mobile, resulting in it being more susceptible to compression from an inflated endotracheal tube (ETT) cuff or traction from surgical retractors. This case illustrates that acute RLNP can rarely occur contralateral to the side of surgical approach in the setting of revision surgery. Surgeons performing revision ACDF can consider approaching from the same side as the index surgery or a posterior approach to reduce the risk of developing bilateral RLNP.

## 1. Introduction

Recurrent laryngeal nerve palsy (RLNP) is a well-recognised complication of anterior cervical spine surgery with an incidence ranging from 0.9% to 8.3% [1]. RLNP usually presents as dysphonia after surgical intervention and has been attributed to intra-operative compression by high endotracheal cuff pressure and/or surgical retractors, traction and less commonly, direct trauma to the nerve [2,3]. Revision surgery has a higher risk of subsequent post-operative RLNP due to the presence of scar tissue and difficulty in dissection [4]. While cases of ipsilateral or even bilateral RLNP post anterior cervical spine surgery have been documented in current literature, to the best of our knowledge, there has been no case report of an isolated RLNP contralateral to the side of the surgical approach following anterior cervical discectomy and fusion (ACDF). We describe a rare case of isolated contralateral RLNP in a patient who underwent revision ACDF for cervical myelopathy and discuss its pathophysiology and implications on the clinical management of patients undergoing revision ACDF.

## 2. Case study

A 72-year-old female presented to the spine surgery clinic following a fall, with a history of progressive gait unsteadiness requiring support for ambulation and bilateral hand numbness over 2 months. 25 years before presentation, she had undergone a C5–C6 ACDF with autologous bone grafting for symptoms of right-sided cervical radiculopathy, which had completely resolved postoperatively. Notably, she did not complain of any post-operative voice abnormalities following her index surgery.

**Fig. 1:**
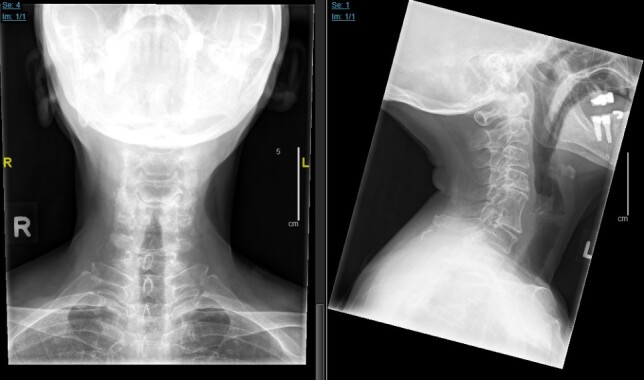
Plain radiographs of the cervical spine demonstrating good bony fusion of C5–C6 and adjacent level degeneration from C3–C7.

**Fig. 2:**
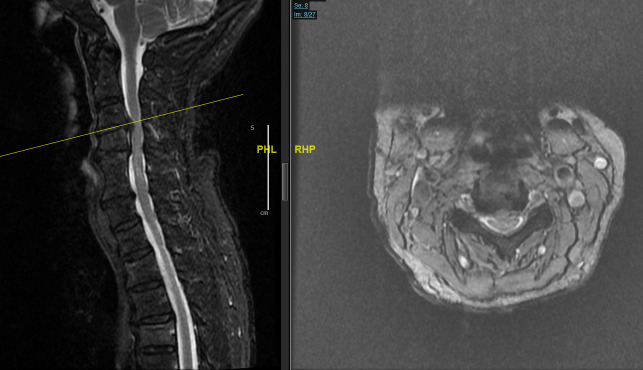
MRI T2-weighted sequences showing multilevel degenerative pathology most significant at C3–C4, where there is severe spinal canal stenosis with cord signal change suggesting myelomalacia.

**Fig. 3:**
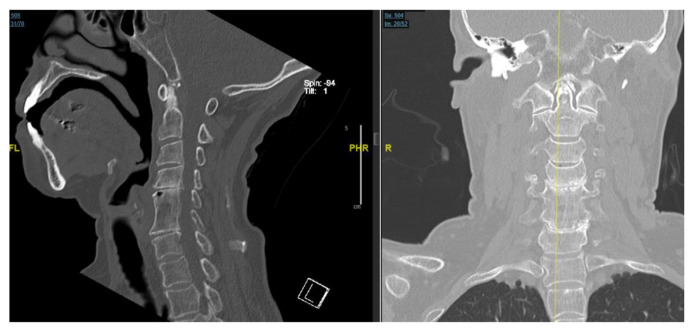
CT cervical spine demonstrating good bony fusion of C5–C6 and adjacent level degeneration from C3–C7.

Clinical examination revealed normal motor power and sensation, with positive signs of cervical myelopathy as evidenced by upper and lower limb hyper-reflexia, positive Hoffman’s sign and inverted supinator reflexes bilaterally. Imaging confirmed the presence of adjacent level degeneration in C3–C5 and C6–C7 levels, with significant spinal canal stenosis and T2-weighted cord signal change most evident at the C3–C4 level. The previously operated level was well fused (Figs. 1–3).

In view of her previous history of anterior cervical spine surgery, a pre-operative otorhinolaryngology consult was obtained to rule out an occult vocal cord palsy. Laryngoscopy revealed normal function of the vocal cords bilaterally.

**Fig. 4:**
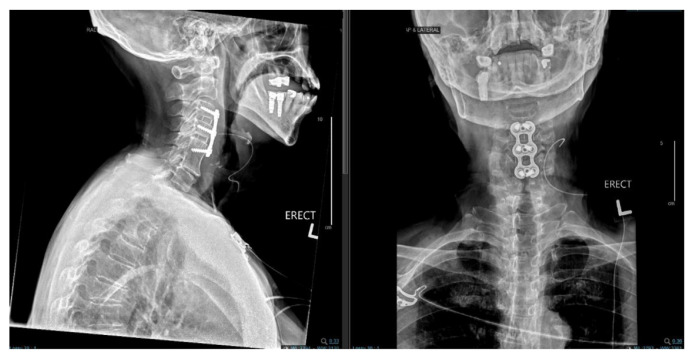
Immediate post op X-rays after C3–C5 ACDF demonstrating satisfactory placement of the implants.

The patient underwent C3–C5 ACDF via a left-sided approach, contralateral to the index surgery to minimise dissection through scar tissue (Fig. 4). Intra-operative findings were that of a large soft central prolapsed intervertebral disc compressing the spinal cord at C3–C4 and severe disc degeneration with reduced disc height at C4–C5. Surgery was uneventful, with an estimated blood loss of 200 ml.

**Fig. 5:**
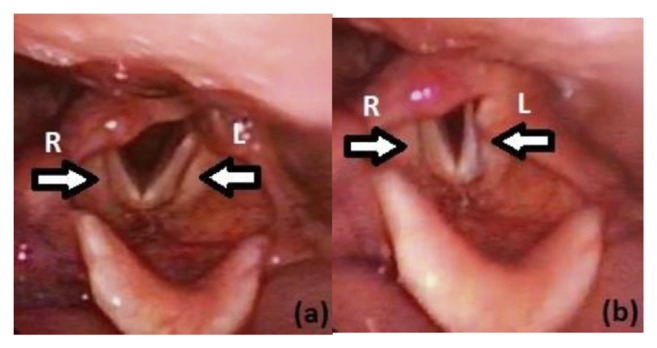
Right vocal cord paralysed in abducted position on phonation, with left vocal cord demonstrating good movement from abduction (a) to adduction (b).

On post-operative day 1, the patient was noted to have marked dysphonia and difficulty tolerating thin fluids and regular diet. Formal otorhinolaryngological assessment by flexible nasoendoscopy revealed a right vocal cord palsy (the side of the previous surgery) in an abducted position on phonation, while the left vocal cord (the acutely operated side) exhibited normal movement (Fig. 5).

The patient was managed conservatively with a 1 week course of dexamethasone, dietary modifications with thickened fluids to prevent aspiration and intensive daily swallowing and voice exercises by a speech therapist. The patient’s dysphagia improved over the course of her admission and did not require nasogastric tube feeding, although her severe dysphonia persisted on discharge.

By 3 months post-operatively, the patient reported that her swallowing dysfunction and dysphonia had resolved. At 1 year, the patient remained satisfied with her post-operative recovery, reporting improvements in her gait disturbances and ambulatory status. She had only minimal voice hoarseness and was able to tolerate a normal diet with normal swallowing function.

## 3. Discussion

RLNP is a well-recognised complication of ACDF [1]. Most commonly, it presents as hoarseness, cough and dysphagia post operatively. Most symptomatic RLNP present immediately post-operatively, although there have been descriptions of delayed presentations [5].

Patients undergoing revision anterior cervical spine surgery are at a higher risk of suffering from RLNP [4]. Bilateral RLNP is a devastating and life-threatening complication that may require emergent airway management post-operatively. It is commonly attributed to surgery performed on the contralateral side of an unrecognised occult vocal cord palsy (e.g. from a previous surgery). Preoperative laryngoscopy has been recommended to reduce such occurrences in this patient group [6].

Various mechanisms underlying the development of RLNP have been proposed by authors, including 1) direct pressure by the inflated cuff of the endotracheal tube (ETT) on the submucosal course of the nerve causing ischemia, 2) traction on the nerve by retraction resulting in neuropraxia, 3) airway trauma during intubation and 4) traumatic division of the nerve [2,3,7]. Unfortunately, as evident from previous studies, it is often difficult to determine the exact mechanism of injury resulting in post-operative RLNP in revision anterior ACDF surgeries [7].

Our patient had a pre-operative laryngoscopy that showed bilateral functional vocal cords, confirming that this complication only occurred during the second surgery. To our knowledge, isolated contralateral RLNP because of anterior cervical spine surgery has not been described in the literature.

With regards to the proposed mechanisms discussed above, it is less likely that a direct injury to the nerve is responsible for the patient’s presentation. Traumatic surgical division of the nerve is not likely as the surgical approach did not extend to the contralateral side. Direct injury during orotracheal intubation can also lead to a vocal cord hematoma resulting in a vocal cord palsy [8]. However, this is unlikely in our patient as a formal postoperative nasoendoscopy did not reveal the presence of a vocal cord hematoma. Anatomical studies have revealed that most probable site of injury is in the subglottic region where the anterior branch of the recurrent laryngeal nerve is most vulnerable to compression between the expanded ETT cuff and overlying thyroid cartilage [9]. We believe that the presence of scar tissue around the contralateral nerve at the previous surgical site has rendered it less mobile, resulting in it being more susceptible to compression and/or traction injuries from the inflated ETT cuff and surgical retractors compared to the ipsilateral side.

Most significantly, this case highlights the possibility that a bilateral vocal cord palsy may still occur in revision anterior cervical spine surgery despite the exclusion of a pre-existing occult RLN palsy on pre-operative laryngoscopy. While this fortunately did not occur to our patient, the possibility of an acute RLNP on the side of the revision surgery combined with a similarly acute contralateral RLNP at the site of the index surgery represents a distinct risk that must be considered.

Intra-operative measures to mitigate the risk of RLNP have been described in the literature, including monitoring of the ETT cuff pressure and intra-operative EMG monitoring of the recurrent laryngeal nerve [10,11], although evidence for these interventions are limited. Neither of these measures were specifically performed for our patient. There are a variety of treatment described for unilateral RLNP [12], fortunately most symptoms are often transient with only a minority of patients experiencing permanent residual symptoms. Majority of patients will experience complete resolution with time [13].

In conclusion, this is a novel case study reporting an isolated RLNP contralateral to the side of the surgical approach in a patient undergoing revision ACDF. It highlights the possibility that despite functional bilateral vocal cords pre-operatively, a bilateral RLNP may potentially occur in patients undergoing revision anterior cervical spine surgery if surgery is performed from the contralateral side to the original procedure. The mechanism behind the development of this complication, in our above case, is likely compression and/or traction to the nerve from the ETT cuff and surgical retractors. Surgeons performing revision anterior cervical spine surgery should be aware of this potential complication and adequately counsel patients on this risk. Using a surgical approach on the same side as the initial surgery or a posterior approach can be considered to reduce the risk of developing a bilateral RLNP.

## Supplementary Information
